# In Vitro Developmental Competence Predicts Pregnancy Outcomes Following Transfer of Beef Embryos to Dairy Recipients: A Retrospective Study

**DOI:** 10.3390/ani16040525

**Published:** 2026-02-07

**Authors:** Sang-Yup Lee, Saet-Byul Kim, Tae-Gyun Kim, Sung-Ho Kim, Seung-Joon Kim, Won-Jae Lee

**Affiliations:** 1 Bovivet, Gumi 39133, Republic of Korea; bovivet@naver.com (S.-Y.L.); bovivet.sbkim@gmail.com (S.-B.K.); 2 College of Veterinary Medicine, Kyungpook National University, Daegu 41566, Republic of Korea; xo1621@naver.com (T.-G.K.); ideat12@knu.ac.kr (S.-H.K.); 3 Institute for Veterinary Biomedical Science, Kyungpook National University, Daegu 41566, Republic of Korea

**Keywords:** embryo developmental competence, embryo transfer, in vitro-produced embryo, pregnancy rate, recipient cattle

## Abstract

In bovine embryo transfer (ET) using in vitro-produced (IVP) embryos, recipient factors and embryo grade are well-established predictors of pregnancy success, but the impact of the laboratory-level developmental competence of IVP embryos remains insufficiently characterized. This retrospective study evaluated factors affecting pregnancy rates following the transfer of IVP beef embryos to dairy recipients. Medical records from 462 ETs were analyzed across three categories: (1) recipient-related factors (parity, body condition, estrus synchronization, corpus luteum characteristics); (2) laboratory factors (cleavage, blastocyst formation, degeneration, embryo grade, developmental stage, cryopreservation); and (3) environmental factors (temperature–humidity index, transport time). Mean comparison and chi-square analyses revealed significant differences in pregnancy rates based on corpus luteum volume, cleavage rates, blastocyst formation rates, degeneration rates, and embryo grade. In binary logistic regression, categorized increases in blastocyst formation rate, degeneration rate, and embryo grade were associated with a 1.45-fold increase, 0.74-fold decrease, and 0.56-fold decrease in pregnancy odds, respectively; no recipient or environmental variables were independent predictors. These findings indicate that developmental competence of IVP embryos is more critical for pregnancy success than recipient or environmental factors, suggesting that optimizing IVP systems to maximize embryo quality is the most effective strategy to improve reproductive efficiency in ET.

## Simple Summary

In cattle breeding, transferring embryos produced in a laboratory into recipient cows is a popular method to improve genetics and beef production efficiency. However, success rates vary, and it is often unclear whether the condition of the recipient cow, the environment, or the quality of the embryo itself is most important for a successful pregnancy. This study analyzed records from 462 transfers of beef cattle embryos into dairy cows to identify the main factors predicting pregnancy success. We examined three categories: the recipient cow’s health and reproductive status, the laboratory performance of the embryos (such as how well they developed in the incubator), and environmental conditions like heat stress. Our results showed that the cow’s condition and weather had little effect on pregnancy outcomes. Instead, the specific developmental ability of the embryos, measured by their growth rates and quality grades in the laboratory, was the strongest predictor of success. Specifically, embryos from groups that grew better and had lower death rates in the lab resulted in significantly higher pregnancy rates. These findings suggest that to improve breeding efficiency, producers should focus primarily on optimizing laboratory systems to produce high-quality embryos rather than focusing solely on managing the recipient cows.

## 1. Introduction

Bovine embryo transfer (ET) and in vitro embryo production have grown rapidly worldwide since the first successful bovine ET in the mid-1900s, with annual production exceeding one million in vitro-produced (IVP) embryos by 2018, marking major progress in reproductive technology [1,2]. ET offers several advantages for cattle breeding, including accelerated genetic improvement, efficient conservation and distribution of elite genetics, facilitated introduction of foreign breeds, and improved biosecurity relative to live animal imports [1,3]. Globally, because of these advantages, a prominent strategy in several countries involves transferring high-value beef embryos into Holstein dairy cows, enabling dairy farmers to generate economically valuable offspring while maintaining milk production in their Holstein herds [4]. Therefore, understanding which factors most strongly influence pregnancy outcomes in beef-on-dairy ET systems is essential for improving reproductive efficiency and farm profitability.

The success of ET relies on complex interactions among embryo, recipient, and environmental factors [3,5]. Embryo-related factors are particularly important, with in vivo-derived (IVD) embryos achieving higher pregnancy rates than IVP embryos [3,4]. Differences in morphology, physiology, and gene expression between IVP and IVD blastocysts impact developmental competence [1,6]. Cryopreservation further decreases viability, as frozen-thawed embryos yield lower conception rates than fresh embryos [4,7]. Embryo quality grade is also essential, with Grade 1 embryos consistently outperforming Grades 2 and 3 [4,6,7]. Recipient-related factors also significantly influence ET success. Among recipient factors, the effect of parity varies; nulliparous heifers tend to do better than lactating cows in dairy breeds, while multiparous cows often perform better in beef breeds [4]. Additional influential factors include corpus luteum (CL) size and progesterone concentration at transfer time, as well as body condition score (BCS), milk yield, and health status [5,7]. Environmental factors, particularly heat stress, can also impact pregnancy outcomes. Heat stress, quantified using the temperature–humidity index (THI), reduces pregnancy rates in lactating cows when THI exceeds 75, although this effect is less pronounced for ET than for artificial insemination (AI) [8].

While recipient factors and embryo morphology (e.g., stage and grade) are well-established predictors of pregnancy success, the impact of laboratory-level developmental competence of IVP embryos on pregnancy outcomes remains insufficiently characterized. Furthermore, comprehensive multifactorial analyses that integrate these established factors with specific developmental competence metrics unique to IVP embryos to quantify their relative contributions remain limited, particularly for IVP beef embryos transferred into dairy recipients under commercial field conditions. Therefore, the objectives of this retrospective study were to: (1) quantify pregnancy outcomes following transfer of IVP beef embryos to dairy recipients under commercial field conditions; (2) evaluate the associations between recipient-related factors (parity, body condition score, estrus synchronization protocol, and corpus luteum characteristics), embryo developmental competence parameters (cleavage rate, blastocyst formation rate, degeneration rate, embryo grade, and developmental stage), and environment-related factors (temperature–humidity index and embryo transport time) with pregnancy rates; (3) identify which factor category independently predicts pregnancy success using binary logistic regression analysis; and (4) provide evidence-based recommendations for optimizing embryo selection strategies and ET program efficiency with IVP embryos.

## 2. Materials and Methods

### 2.1. Ethical Statements

All experimental procedures in animals were approved by the Institutional Animal Care and Use Committee at Kyungpook National University (approval number: 2025-0180).

### 2.2. Chemicals and Media

Unless otherwise specified, all chemicals and reagents used for IVP embryo production were purchased from Thermo Fisher Scientific (Waltham, MA, USA).

### 2.3. Study Location and Animals

From January to September 2025, medical records related to ET in Holstein cows (*n* = 462) from 14 dairy farms near a private research institute located near Gumi, Korea (latitude: 36°7′ N, longitude: 128°20′ E), were retrospectively analyzed. This included 250 nulliparous heifers (about 15 months old) and 212 lactating cows (at least 60 days postpartum) as recipients. For lactating cows, TMR feed was provided so that they could have constant access to a sufficient amount of feed, with the remaining feed in the trough adjusted to about 5% at the next day’s feeding. For heifers, hay and concentrate were provided twice daily according to the standards of the Korean National Institute of Animal Science. Mineral blocks and water were also supplied ad libitum. Only recipients for whom ovarian cyclicity, defined as the regular and repeated pattern of follicular development, ovulation, and CL formation and regression, as confirmed prior to estrus synchronization by transrectal ultrasonography using a 7.5 MHz linear probe (Easy-Scan Go, IMV, Bellshill, Scotland, UK), were included. The mean BCS was 3.2 (range: 2.5–4.25), ensuring exclusion of nutritionally deficient animals likely to affect pregnancy rates. Estrus synchronization, CL evaluation, and ET for this retrospective analysis were performed by two veterinarians with more than 5 years of clinical experience.

### 2.4. Preparation of In Vitro-Produced Bovine Embryos for Embryo Transfer

The ovaries from Hanwoo (Korean native beef cow, *Bos taurus coreanae*) for IVP were collected from a local abattoir and transported at 37.0 °C. As described in previous articles [6,9], cumulus–oocyte complexes (COCs) were aspirated from 3 to 10 mm follicles, and only those with compact cumulus, homogeneous cytoplasm, and uniform ooplasm were selected for in vitro maturation in TCM-199 medium supplemented with hormones, fetal calf serum, pyruvate, and gentamycin at 38.5–39.0 °C for 22–24 h under humidified 5% CO_2_. For in vitro fertilization, motile sperm from frozen–thawed semen were isolated using the swim-up method. Ten mature COCs were inseminated in TALP medium containing heparin at 2 × 10^6^ sperm/mL and incubated at 38.5–39.0 °C for 18–20 h; the time immediately after insemination was defined as the starting point for hours post-insemination (0 hpi). The presumptive zygotes were denuded and cultured in SOF medium with albumin and gentamycin under a gas mixture (5% CO_2_, 5% O_2_, and 90% N_2_) in a multi-gas incubator. At 72–74 hpi, cleaved embryos (≥2 cells) were selected and transferred to fresh medium, and development was monitored daily until 144–168 hpi; degenerated embryos were discarded. Cleavage, blastocyst formation, and degeneration rates were calculated as percentages of total presumptive zygotes. Procedures were repeated for 35 IVP production batches; to exclude laboratory-derived variation, all IVP procedures were performed in the same laboratory under tightly controlled culture conditions, with incubator gas composition checked at least weekly using an external gas analyzer, culture temperature routinely verified with a calibrated mercury thermometer to maintain 38.5–39.0 °C, and culture medium pH monitored during embryo culture and kept at 7.2 ± 0.1 by adjusting gas settings. According to the International Embryo Technology Society’s criteria, embryos at 144–168 hpi were classified by developmental stage as morula (Stage 3), compact morula (Stage 4), early blastocyst (Stage 5), blastocyst (Stage 6), expanded blastocyst (Stage 7), or hatched blastocyst (Stage 8), and by quality grade as Grade 1 (excellent or good), Grade 2 (fair), or Grade 3 (poor). In the embryos used in this study, Grade 1 embryos tended to progress through developmental stages more uniformly than lower-grade embryos (Stages 3–4: 25.6%; Stages 5–6: 27.9%; Stages 7–8: 46.6%), whereas lower-grade embryos showed a tendency to remain at earlier stages (Stages 3–4: 70.3% and 57.5% for Grades 2 and 3, respectively; Table 1). In this study, Grades 1–3 embryos were transferred fresh, while some Grade 1 blastocysts were vitrified using the Kitazato Vitrification Kit and Cryotop^®^ (Kitazato Corporation, Fuji, Shizuoka, Japan). Only morphologically intact, viable embryos were transferred after warming.

### 2.5. Estrus Synchronization

Estrus synchronization was achieved by either natural estrus (Natural) detection or induced ovulation. Ovulation induction was selected based on CL maturity and follicular status, specifically whether follicular selection or atresia was occurring, using two protocols. The prostaglandin F_2α_ (PGF_2α_) and gonadotropin-releasing hormone (GnRH) protocol (PG+GN) was applied primarily when a dominant follicle was expected to emerge 2–3 days after initial PGF_2α_ administration. A PGF_2α_ analog (Repromate, Unibiotech Co., Ltd., Anyang, Republic of Korea) was administered, followed by a GnRH analog (Buserelin, Unibiotech Co., Ltd.) 2.5 days later. The Ovsynch protocol was used on Days 5–9 post-natural estrus. The first GnRH injection was followed by PGF_2α_ 7 days later and a second GnRH injection 2.5 days afterward. For both protocols, the timing of the final GnRH injection defined Day 0 of CL age. On the day before ET (CL age: 6.0–6.5 days), transrectal ultrasonography was used to assess CL characteristics, including location (left, right, or bilateral), number (single or double), presence of cavitary CL (a normal developmental variant on Day 6 of CL age rather than a pathological cystic CL), dominant follicle diameter (cm), and CL diameter (cm). CL and dominant follicle volumes were calculated using the sphere volume formula (4/3πr^3^, where r was determined from measured diameters). In cases of cavitary CL, actual luteal tissue volume was calculated by subtracting cavity volume from total CL volume.

### 2.6. Embryo Transfer, Temperature–Humidity Index, and Pregnancy Diagnosis

Embryos were transferred in batches of 3 to 20 per day, with a single embryo transferred to each recipient. IVP embryos were loaded into 0.25 mL straws (IMV Technologies, L’Aigle, France) containing culture medium, sealed, and transported horizontally in a thermos at 38 °C; transport time was recorded. At Days 7–7.5 of CL age, embryos were transferred non-surgically to the uterine horn ipsilateral to the CL, depositing them at the most distal portion of the horn using a standard ET gun. THI on the transfer day was calculated using data from the Livestock Meteorological Information System of the Rural Development Administration of the Republic of Korea and the dairy cattle formula: THI = (1.8 × temperature + 32) − [(0.55 − 0.0055 × relative humidity) × (1.8 × temperature − 26.8)]. THI values were categorized as follows: <72 (normal, optimal), 73–77 (caution, normal but borderline), 78–88 (alert, reduced feed intake and milk yield), 89–97 (danger, marked reduction in feed intake and milk yield), and ≥98 (emergency, risk of mortality). Although the study period included the midsummer season, there were no days on which THI exceeded 89; therefore, in practice, only three THI categories (≤72, 73–77, and 78–88) were used for statistical analysis of pregnancy rates. Pregnancy diagnosis was performed by transrectal ultrasonography at approximately 30 and 90 days post-ET to determine initial pregnancy status (presence of embryo and amniotic vesicle) and monitor pregnancy maintenance (fetal viability and placental development), respectively. Recipients exhibiting embryonic loss, resorption, or abortion between the two examinations were classified as non-pregnant and excluded from the final pregnancy rate analysis.

### 2.7. Statistical Analysis

This retrospective analysis utilized medical records collected during routine ET procedures. Statistical analyses were performed using SPSS software (version 12.0, IBM Corporation, Armonk, NY, USA). In this retrospective analysis, our statistical approach addressed two distinct research questions through complementary analyses. (1) To investigate whether pregnancy rates differ on average across specific factors, we calculated pregnancy rates for each IVP batch within each factor category and performed mean comparisons using ANOVA. To ensure statistical normality and homogeneity of variance, batches that included only 1–2 recipients within a given factor category were excluded from the ANOVA, because such small groups can produce extreme values (0% or 100%) that do not satisfy the assumptions of parametric tests. (2) To evaluate whether the frequency distribution of pregnancy versus non-pregnancy differed among factor categories and to identify which factors independently predicted pregnancy outcomes, we performed analyses at the individual ET level (*n* = 462) using chi-square tests and binary logistic regression. Continuous variables are expressed as mean ± standard error of the mean values and were compared using ANOVA with Duncan’s post hoc test or Student’s *t*-test to evaluate differences in mean pregnancy rates across factor categories. Continuous variables were converted into categorical variables based on the criteria defined in Table 2, Table 3 and Table 4 to facilitate cross-tabulation and binary logistic regression analyses; of note, higher grade numbers indicate lower embryo quality, such that Grade 1 embryos (categorical variable: 1) represent the highest quality (excellent or good), while Grade 3 embryos (categorical variable: 3) represent the lowest quality (poor). For categorical variables, pregnancy rates across in vivo recipient-related, in vitro laboratory, and environmental factor groups were compared using cross-tabulation analysis with chi-square tests to assess associations between categorized factors and pregnancy outcomes (pregnant vs. non-pregnant). To identify independent predictors of pregnancy success while controlling for potential confounding effects, multivariable binary logistic regression analysis was performed with pregnancy outcome (pregnant vs. non-pregnant) as the dependent variable. All variables from recipient-related, laboratory-related, and environmental factor categories were entered simultaneously into the model using the Enter method, allowing each variable to be adjusted for all other variables in the model. Odds ratios (OR) with 95% confidence intervals were calculated to quantify the strength of independent associations between predictors and pregnancy outcomes. Statistical significance was set at *p* < 0.05 for all analyses.

## 3. Results

### 3.1. Embryo Development and Overall Pregnancy Outcomes

**Figure 1:**
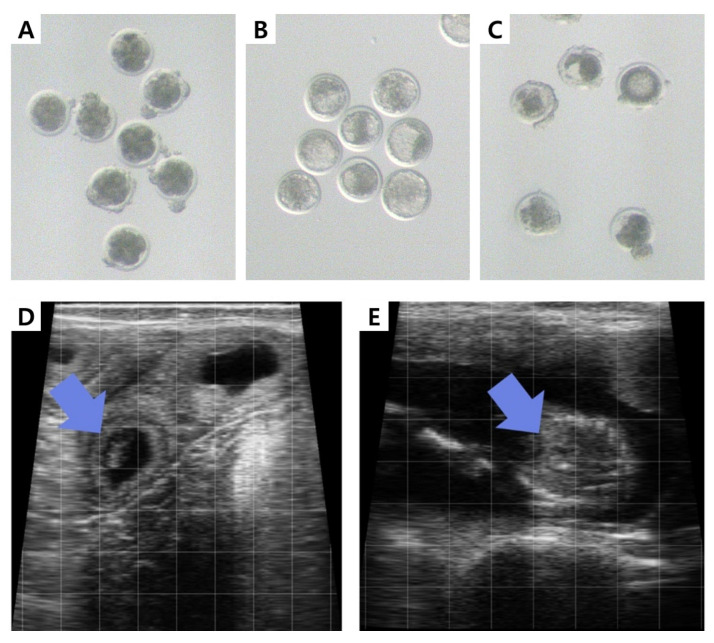
Representative images for embryo developmental competence and pregnancy outcomes. (**A**) Cleaved embryos were selected at 72–74 hpi. (**B**) At 144–168 hpi, various stages (3–6) and grades (1–3) of embryos were transferred to recipient cows. (**C**) Degenerated embryos were discarded during in vitro culture. (**D**,**E**) Pregnancy diagnosis was performed by transrectal ultrasonography at approximately 30 and 90 days post-ET to determine initial pregnancy status (presence of embryo and amniotic vesicle indicated by a blue arrow in panel (**D**)) and monitor pregnancy maintenance (fetal viability and placental development indicated by a blue arrow in panel (**E**)), respectively.

The IVP embryos used in this study exhibited developmental performance (Figure 1A–C) values of 84.1 ± 5.8% cleavage, 31.7 ± 9.7% blastocyst formation, and 23.9 ± 7.9% degeneration, indicating the transfer of relatively uniform IVP embryos into Holstein recipients. Overall pregnancy diagnosis (Figure 1D,E) at 30 days post-transfer yielded a pregnancy rate of 47.0% (217/462), which declined to 43.9% (203/462) by day 90. The pregnancy maintenance rate from Day 30 to Day 90 was 93.5% (203/217), corresponding to an embryo loss rate of 6.5% (14/217).

### 3.2. Recipient Factors and Pregnancy Outcomes

**Figure 2:**
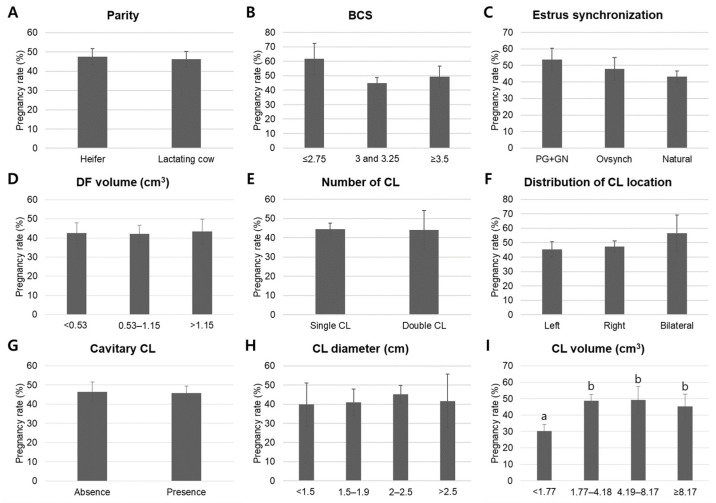
Mean comparison analysis of in vivo recipient-related factors influencing pregnancy rate following embryo transfer. Pregnancy rates were analyzed based on parity (**A**), BCS (**B**), estrus synchronization method (**C**), and CL characteristics on the day before transfer, including DF volume (**D**), number of CL (**E**), CL location in the ovary (**F**), presence of cavitary CL (**G**), CL diameter (**H**), and CL volume (**I**). Data are presented as mean ± standard error of the mean (SEM). Different letters indicate significant differences among groups (*p* < 0.05). Abbreviation: BCS, body condition score; PG+GN, prostaglandin F_2α_ and gonadotropin-releasing hormone protocol; DF, dominant follicle; CL, corpus luteum.

Retrospective evaluation using mean comparison analysis with ANOVA and Duncan’s post hoc test (Figure 2) and cross-tabulation analysis with chi-square tests (Table 2) examined the effects of recipient-related factors on pregnancy outcomes. Neither recipient parity (nulliparous heifers: 47.5 ± 4.3%; postpartum lactating cows: 46.2 ± 4.1%) nor BCS (BCS ≤ 2.75: 61.7 ± 10.9%; BCS 3.0 to 3.25: 44.9 ± 4.0%; BCS ≥ 3.5: 49.4 ± 7.2%) demonstrated significant differences among groups by ANOVA, consistent with chi-square test results in cross-tabulation analysis. Although induced estrus synchronization groups showed numerically higher pregnancy rates than the natural estrus group (PG+GN: 53.5 ± 6.8%; Ovsynch: 47.8 ± 6.9%; Natural: 43.1 ± 3.5%), these differences were not statistically significant in either analytical approach.

In the case of CL characteristics, mean comparison analysis using ANOVA with Duncan’s post hoc test revealed that while CL location, CL number, presence of a cavitary CL, CL diameter, and dominant follicle diameter were not associated with pregnancy outcome, CL volume categories exhibited significantly different mean pregnancy rates (*p* < 0.05, Figure 2I). Specifically, recipients with CL volumes of 1.77 to 4.18 cm^3^, 4.19 to 8.17 cm^3^, and ≥8.17 cm^3^ had higher mean pregnancy rates (48.7 ± 3.9%, 49.3 ± 8.1%, and 45.3 ± 7.5%, respectively) compared with those with volumes < 1.77 cm^3^ (30.3 ± 3.9%). Cross-tabulation analysis with chi-square tests (Table 2) demonstrated a trend toward statistical significance for CL volume (*p* = 0.078). However, in binary logistic regression, CL volume did not emerge as an independent predictor of pregnancy (*p* = 0.524), indicating that its effect depends on other factors (Table 5).

### 3.3. Laboratory Factors and Pregnancy Outcomes

**Figure 3:**
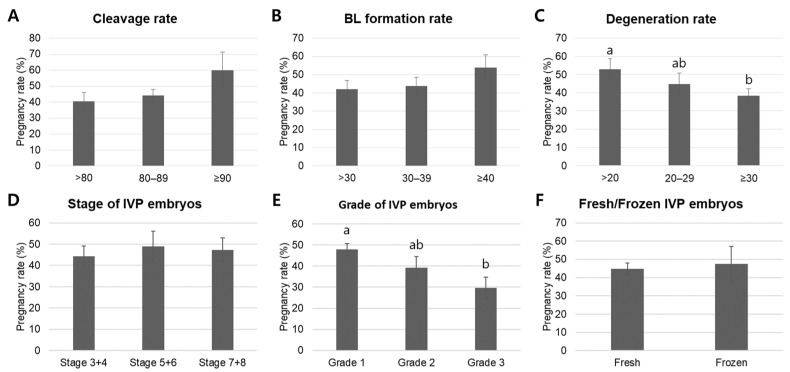
Mean comparison analysis of in vitro laboratory factors influencing pregnancy rate following embryo transfer. Pregnancy rates were analyzed based on cleavage rate (**A**), blastocyst formation rate (**B**), degeneration rate (**C**), embryo stage (**D**), embryo grade (**E**), and cryopreservation status (**F**). Data are presented as mean ± SEM. Different letters indicate significant differences among groups (*p* < 0.05). Abbreviation: IVP, in vitro-produced; BL, blastocyst.

Retrospective examination of IVP embryo production records and their associated pregnancy outcomes following ET was conducted to evaluate developmental competence parameters. Mean comparison analysis using ANOVA with Duncan’s post hoc test (Figure 3) revealed that embryos from batches with higher cleavage rates (>80%: 40.5 ± 5.3%, 80–89%: 44.0 ± 3.9%, ≥90%: 60.0 ± 11.2%) and higher blastocyst formation rates (>30%: 42.0 ± 4.8%, 30–39%: 43.7 ± 4.8%, ≥40%: 53.8 ± 7.0%) yielded increased mean pregnancy rates, although the differences among groups were not statistically significant according to ANOVA. However, cross-tabulation analysis with chi-square tests (Table 3) demonstrated significant differences (*p* = 0.017) in pregnancy rates according to blastocyst formation categories. The degeneration rate during embryo production significantly affected pregnancy outcomes in both mean comparison analysis (ANOVA with Duncan’s post hoc test) and cross-tabulation analysis (chi-square test). Batches with higher degeneration rates exhibited significantly (*p* < 0.05) reduced mean pregnancy rates according to ANOVA (>20%: 53.0 ± 5.6%, 20–29%: 44.7 ± 6.2%, ≥30%: 38.4 ± 3.7%). Regarding embryo developmental stage, pregnancy rates for Stage 3 + 4 (44.4 ± 4.8%), Stage 5 + 6 (49.0 ± 7.15%), and Stage 7 + 8 (47.3 ± 5.59%) did not differ significantly among groups. However, embryo grade significantly (*p* < 0.05) influenced mean pregnancy rates according to ANOVA with Duncan’s post hoc test, with pregnancy rates proportionally increasing with embryo quality: Grade 1 (47.8 ± 2.9%), Grade 2 (39.3 ± 5.2%), and Grade 3 (29.6 ± 5.1%). No significant difference in mean pregnancy rates was observed between fresh (44.7 ± 3.4%) and frozen–thawed (47.4 ± 9.8%) Grade 1 embryos according to Student’s *t*-test at the time of ET. Binary logistic regression analysis revealed patterns consistent with the significant differences observed in mean comparison and cross-tabulation analyses (Table 5). It identified blastocyst formation rate, degeneration rate, and embryo grade as independent predictors. For each incremental increase in group classification level, the OR increased 1.45-fold for blastocyst formation rate (*p* = 0.022), decreased 0.74-fold for degeneration rate (*p* = 0.013), and declined 0.56-fold for embryo grade (*p* = 0.002).

### 3.4. Environmental Factors and Pregnancy Outcomes

**Figure 4:**
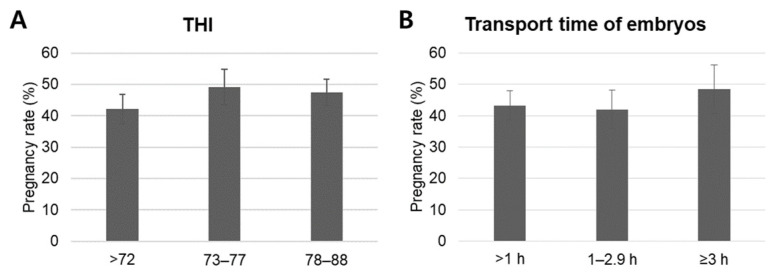
Mean comparison analysis of environmental factors influencing pregnancy rate following ET. Pregnancy rates were analyzed based on THI (**A**) and the transport time (from laboratory to farm) of embryos for ET (**B**). Data are presented as mean ± SEM. Abbreviations: THI, temperature–humidity index; ET, embryo transfer.

In addition to recipient-related factors and in vitro laboratory factors, the effects of environmental factors, specifically THI and embryo transport time for ET, were retrospectively evaluated. In the study region, multiple IVP batches were transferred weekly, including during summer; however, no ET procedures were performed on days when THI exceeded 89 (danger) and 98 (emergency). Mean comparison analysis using ANOVA with Duncan’s post hoc test (Figure 4) revealed that mean pregnancy rates did not differ significantly across transport-time categories (>1 h: 43.3 ± 4.7%, 1–2.9 h: 42.0 ± 6.1%, ≥3 h: 48.4 ± 7.9%) or THI categories (≤72: 42.1 ± 4.6%, 73–77: 49.1 ± 5.7%, 78–88: 47.5 ± 4.2%). Cross-tabulation analysis with chi-square tests (Table 4) yielded consistent results, demonstrating no significant associations between these environmental factors and pregnancy outcomes. Binary logistic regression further confirmed that neither embryo transport time nor THI functioned as independent predictors of pregnancy rate (Table 5).

## 4. Discussion

ET technology is essential for cattle breeding, with IVP embryos enabling efficient genetic dissemination and allowing dairy farmers to produce economically valuable offspring while maintaining Holstein herd milk production [3,4]. Through retrospective analysis of 462 ET cases, this study comprehensively evaluated recipient-, laboratory-, and environmental-related factors and found that laboratory factors were more closely associated with pregnancy rates than other categories in beef-on-dairy ET systems using IVP embryos.

In the present study, the observed embryo loss rate of 6.5% between day 30 and day 90 post-transfer was lower than the ranges previously reported for IVP embryos (7.7–10.0%) [5,10]. It has been demonstrated that pregnancies confirmed at day 75 proceeded to parturition without further loss (0.0% loss from day 75 to calving), and a recent meta-analysis reported that approximately 98% of pregnancies confirmed at day 60 in dairy cattle are maintained to parturition [5,11]. By extrapolation, the recipients confirmed pregnant at day 90 in the present study are expected to achieve high calving success, indicating that the ET system employed in this study is comparatively stable and well-optimized.

The effects of parity, BCS, and estrus synchronization protocol on pregnancy rates after ET have been evaluated extensively, yet consistent conclusions have not been reached. Some studies report that Holstein heifers (34.2–55.1%) have significantly higher pregnancy rates than multiparous cows (12.2–45.6%), largely due to metabolic stress from lactation, which adversely affects embryo survival [4,12,13,14]. However, other large-scale studies have found no clear parity effect, showing comparable pregnancy rates between dairy heifers (50.6–56.3%) and cows (42.0–58.8%), and in some cases even reporting higher rates in multiparous cows [7,15,16]. For BCS, no significant difference was observed among adequately managed recipients in this study, and analyses comparing animals below and above the 2.75 BCS threshold revealed no statistical significance [17,18]. Synchronization protocol alone was also not predictive for ET success, with comparable pregnancy rates observed between Ovsynch+CIDR^®^ and single PGF_2α_ in beef cows and between natural estrus and Ovsynch in Holsteins [4,19]. In this retrospective analysis, parity, BCS, and synchronization method were not significant predictors of pregnancy rate, indicating that ET success is multifactorial rather than dependent on any single recipient-related factor.

In previous studies, highly variable and often contradictory results have been reported regarding which CL characteristics most reliably predict ET success. Some reports indicate that CL structural classification and texture influence pregnancy rates, with asymmetric and soft-textured small CLs demonstrating significantly lower success than firm and high-quality CLs with diameters greater than 20 mm (16.3–31.7% vs. 31.0–46.7%) [4,20,21]. Conversely, other studies evaluating CL size, crown prominence, morphology, and serum progesterone concentrations have found no differences in pregnancy rate across CL grades [5,22,23]. In this study, CL location, number, presence of a cavitary CL, CL diameter, and dominant follicle diameter were not significantly associated with pregnancy outcome, whereas a CL volume below 1.77 cm^3^ resulted in a significantly lower pregnancy rate. However, logistic regression analysis indicated that CL volume was not an independent predictor of pregnancy, which agrees with previous reports [19,20,24,25]. The observed discrepancy between univariate and multivariate analyses regarding CL volume may be explained by confounding effects of embryo developmental competence. While CL volume serves as a proxy for progesterone production and uterine environment quality, its predictive value diminishes when embryo quality variables (blastocyst formation rate, degeneration rate, and embryo grade) are included in the model. This suggests that embryo developmental competence exerts a more direct and dominant influence on pregnancy establishment than recipient CL characteristics. These findings suggest that although smaller CL volumes may negatively impact pregnancy success in univariate analysis, their predictive strength diminishes in multivariate models. The presence and size of the CL central cavity have also shown inconsistent outcomes. Some reports observed no differences in pregnancy rates based on the cavity diameter-to-CL diameter ratio [23], whereas others reported a positive effect of cavitary CLs in ET of IVP embryos [24]. These conflicting findings highlight the need for further research to determine which CL factors best predict ET success. Given that pregnancy rates were significantly lower for recipients with small CL volumes, this retrospective analysis suggests that CL volume is a more comprehensive indicator for pregnancy rate than CL diameter alone; however, complex interactions among CL characteristics, embryo quality, and recipient factors limit the predictive value of any single CL variable for pregnancy outcomes. These findings underscore a hierarchical relationship: while adequate CL function (reflected in CL volume) provides a permissive uterine environment, the intrinsic developmental competence of the embryo ultimately determines pregnancy outcome. This interpretation aligns with the established reproductive physiology principle that embryo quality is the rate-limiting factor in pregnancy establishment when recipient animals meet minimum thresholds for reproductive competence.

Previous studies have reported wide variations in the effects of embryo characteristics on pregnancy outcomes, with results differing between IVP and IVD embryos. In IVD embryo research, some studies reported significant stage-dependent differences in pregnancy rates (46.3–64.9% for stage 5–6 embryos vs. 37.3–64.1% for stage 4 embryos and 33.3–62.4% for stage 7 embryos) [10,16,26]. Other reports found no differences across embryo stages (32.3–72.7% from stage 4–7 embryos) [5,27]. In IVP embryo research, this study’s findings for IVP embryos are consistent with previous IVP analyses, showing no significant effect of embryo stage on pregnancy rate [28]. Conversely, embryo quality grade was identified as the most influential determinant of pregnancy success in both IVP and IVD embryos. In this study, Grade 1 IVP embryos achieved a pregnancy rate of 47.8%, and Grades 2–3 yielded a pregnancy rate of 39.3–29.6%, matching previous reports (Grade 1: 51.0–73.2%; Grades 2–3: 17.8–63.6%) [6,25,29]. And analyses revealed IVP embryo grade to be an independent predictor for ET success, with lower grades associated with a 0.56-fold reduction in pregnancy rate. IVD studies also consistently reported higher pregnancy rates for Grade 1 embryos (40.7–66.2%) compared with Grade 2 (27.7–62.2%) and Grade 3 (36.8–52.6%) [7,10,11,13]. Consistent with previous findings, we observed no significant difference in pregnancy rates between fresh embryos (44.7%) and frozen–thawed (47.4%) Grade 1 IVP embryos, confirming that high-quality IVP embryos possess sufficient cryotolerance [28]. Overall, this retrospective study emphasizes that embryo quality grade is an independent predictor of pregnancy rate in IVP embryos, highlighting the need to prioritize high-quality IVP embryos for transfer and cryopreservation in ET programs.

This study demonstrated that the cleavage rate, blastocyst formation rate, and degeneration rate of IVP embryos are important predictors of pregnancy success after ET, and these parameters closely reflect the cellular and molecular quality of IVP embryos. Previous studies indicate that a high blastocyst formation rate, identified as an independent predictor in this study, is associated with increased amino acid and citric acid utilization, enhanced ATP synthesis, greater oxidative phosphorylation activity, and improved mitochondrial function, all of which contribute to higher metabolic efficiency and pregnancy rates [30,31,32]. Previously, we also found that high-quality embryos demonstrated reduced apoptosis, increased expression of pluripotency-related genes (*Oct4* and *Sox2*), and higher inner cell mass ratios, all of which support successful pregnancy [6,9]. Another independent predictor, a high degeneration rate, likely impairs developmental and pregnancy competence by increasing oxidative stress, cellular senescence, and apoptosis [6,33,34,35]. Collectively, these developmental parameters function as integrated indicators of metabolic activity, stress resistance, and cellular homeostasis, and determine an embryo’s ability to establish and maintain pregnancy after transfer. These findings demonstrate that, in bovine ET programs, selecting embryos based on IVP developmental metrics enables more accurate prediction and improvement of pregnancy outcomes. Importantly, given that such high-quality blastocysts can be sufficiently increased through laboratory interventions, optimizing in vitro embryo production protocols is central for successful bovine ET outcomes [36].

Although AI pregnancy rates in dairy cows decline sharply during summer, many studies report that ET exhibits minimal seasonal variation and can overcome the negative effects of heat stress. Specifically, ET pregnancy rates remain stable up to a THI of 60 to 70, and even when THI exceeds 76–80, only mild or moderate decreases have been observed [4,10,14]. In this retrospective study’s region, ET was not performed on days when THI exceeded 89; therefore, the effect of ambient heat stress on pregnancy outcomes was limited. However, as global warming continues, THI may become an independent predictor of ET success in the future research.

Several limitations of this study should be acknowledged, which restrict the generalizability of our conclusions regarding the relative importance of recipient, laboratory, and environmental factors. As a retrospective study with sample size determined by available cases rather than a priori power calculations, smaller effect sizes may not have been reliably detected, which should be considered when interpreting non-significant trends such as CL volume (*p* = 0.078, Table 2) and cleavage rate (*p* = 0.093, Table 3). In addition, the study population was relatively controlled, including only recipients with normal ovarian cyclicity, a narrow BCS range (2.5–4.25), and moderate ages, so animals with extreme body condition, metabolic disease, reproductive abnormalities, or poor health were not represented; in such populations, recipient factors might have a much greater impact than observed here. All IVP embryos were produced from Hanwoo oocytes in a single laboratory, so differences among laboratories, breeds, or culture systems could lead to different patterns of embryo developmental competence. Environmental conditions were also restricted to one region in Korea, where THI values did not exceed 89 during the study period, limiting assessment of severe environmental stress. Furthermore, unmeasured confounders such as individual health history, farm-specific management practices, or nutritional programs, and the fact that all ETs were performed by two veterinarians at 14 farms may limit extrapolation to other systems. Regarding cryopreservation, this study found no significant difference in pregnancy rates between fresh (44.7%) and frozen–thawed (47.4%) Grade 1 IVP embryos, indicating that high-quality IVP embryos have sufficient cryotolerance; however, only Grade 1 blastocysts were vitrified, consistent with commercial practice in which Grade 1 embryos are preferentially selected for cryopreservation [4,6,7], so these results cannot be generalized to lower-quality (Grade 2 or 3) embryos, which may be more susceptible to cryoinjury, and comparative data for fresh versus frozen–thawed Grade 2 or Grade 3 embryos are not available. Taken together, these limitations indicate that our findings apply specifically to well-managed commercial ET programs operating under controlled conditions similar to those in this study, where embryo developmental competence appears to be the rate-limiting factor; in settings with greater variability in recipient health, more extreme environmental conditions, or different IVP systems, the relative importance of recipient, laboratory, and environmental factors may differ.

## 5. Conclusions

Previous studies have indicated that ET pregnancy rates in dairy cattle can vary depending on which influencing factors are present and how they interact. In this study, a retrospective analysis of 462 ET procedures performed under well-managed commercial conditions (normal BCS, confirmed normal ovarian cyclicity, and moderate environmental conditions) showed that laboratory factors, such as blastocyst formation rate, degeneration rate, and embryo grade, were the main independent predictors of pregnancy success following transfer of Hanwoo embryos into Holstein recipients, whereas recipient and environmental factors within these optimized ranges did not emerge as independent predictors. These findings suggest that when recipients meet minimum reproductive competence and environmental conditions remain within acceptable limits, embryo developmental competence becomes the rate-limiting factor for pregnancy establishment, although in populations with extreme body condition, metabolic disease, reproductive abnormalities, or severe environmental stress, recipient and environmental factors may have a much stronger impact on pregnancy outcomes. Therefore, under well-managed conditions in ET programs using IVP embryos, improving pregnancy performance depends primarily on optimizing embryo production protocols to enhance embryo quality, supported by appropriate recipient and environmental management, and this study provides practical insights for building efficient laboratory management and embryo evaluation systems under such controlled commercial conditions.

## Figures and Tables

**Table 1:** Distribution of embryo grade and developmental stage.

Embryo Grade	Stage 3–4, *n* (%) *	Stage 5–6, *n* (%) *	Stages 7–8, *n* (%) *
Grade 1	89 (25.6)	97 (27.9)	162 (46.6)
Grade 2	52 (70.3)	14 (18.9)	8 (10.8)
Grade 3	23 (57.5)	12 (30.0)	5 (12.5)

* *n* (%) indicates the number and corresponding percentage of embryos at each developmental stage within a given grade category.

**Table 2:** Effect of recipient-related factors on pregnancy rate following IVP beef embryo transfer to dairy cattle.

Factor	Categorization Criteria	Categorized Variable	Pregnancy Rate (%) *	Χ^2^	*p*-Value
Parity	Heifer	1	43.6 (109/250)	0.251	0.880
	Lactating cow	2	44.3 (94/212)		
BCS	≤2.75	1	53.1 (26/49)	2.283	0.319
	3 and 3.25	2	41.8 (123/294)		
	≥3.5	3	45.4 (54/119)		
Estrus synchronization	PG+GN	1	47.4 (54/114)	0.994	0.608
	Ovsynch	2	45.0 (45/100)		
	Natural	3	41.9 (104/248)		
DF volume (cm^3^)	<0.53	1	43.9 (79/180)	2.647	0.266
	0.53–1.15	2	40.4 (74/183)		
	>1.15	3	50.5 (50/99)		
Number of CL	Single CL	1	43.9 (182/415)	0.002	0.961
	Double CL	2	44.6 (21/47)		
CL location in the ovary	Left	1	42.5 (71/167)	0.689	0.709
	Right	2	44.1 (116/263)		
	Bilateral	3	50.0 (16/32)		
Cavitary CL	Absence	1	46.2 (60/130)	0.268	0.605
	Presence	2	43.1 (143/332)		
CL diameter (cm)	<1.5	1	41.2 (7/17)	1.313	0.726
	1.5–1.9	2	41.1 (51/124)		
	2–2.5	3	46.5 (112/241)		
	>2.5	4	41.3 (33/80)		
CL volume (cm^3^)	<1.77	1	31.3 (21/67)	2.423	0.078
	1.77–4.18	2	47.3 (124/262)		
	4.19–8.17	3	44.7 (34/76)		
	≥8.17	4	42.1 (24/57)		

* Data are presented as pregnancy rate (number of pregnant cows/total number of cows) of each factor. Statistical analysis: Chi-square test. Abbreviations: BCS, body condition score; DF, dominant follicle; CL, corpus luteum.

**Table 3:** Effect of in vitro laboratory factors on pregnancy rate following IVP beef embryo transfer to dairy cattle.

Factor	Categorization Criteria	Categorized Variable	Pregnancy Rate (%) *	Χ^2^	*p*-Value
Cleavage rate of embryos	>80%	1	40.0 (36/90)	4.752	0.093
	80–89%	2	42.5 (131/308)		
	≥90%	3	56.3 (36/64)		
BL formation rate of embryos	>30%	1	36.3 (62/171)	8.113	0.017
	30–39%	2	45.6 (82/180)		
	≥40%	3	53.2 (59/111)		
Degeneration rate of embryos	>20%	1	49.0 (97/198)	6.016	0.049
	20–29%	2	47.1 (40/85)		
	≥30%	3	36.9 (66/179)		
Stage of IVP embryos	Stages 3 and 4	1	42.7 (70/164)	0.634	0.728
	Stages 5 and 6	2	42.3 (52/123)		
	Stages 7 and 8	3	46.3 (81/175)		
Grade of IVP embryos	Grade 1	1	47.7 (166/348)	8.716	0.013
	Grade 2	2	35.1 (26/74)		
	Grade 3	3	27.5 (11/40)		
Fresh/Frozen IVP embryos	Fresh	1	43.3 (178/411)	0.601	0.438
	Frozen	2	49.0 (25/51)		

* Data are presented as pregnancy rate (number of pregnant cows/total number of cows) of each factor. Statistical analysis: Chi-square test. Abbreviations: IVP, in vitro-produced; BL, blastocyst.

**Table 4:** Effect of environmental factors on pregnancy rate following IVP beef embryo transfer to dairy cattle.

Factor	Categorization Criteria	Categorized Variable	Pregnancy Rate (%) *	Χ^2^	*p*-Value
THI	>72	1	40.2 (80/199)	2.012	0.366
	73–77	2	45.9 (34/74)		
	78–88	3	47.1 (89/189)		
Transport time of embryos	>1 h	1	43.5 (110/253)	0.117	0.943
	1–2.9 h	2	43.9 (65/148)		
	≥3 h	3	45.9 (28/61)		

* Data are presented as pregnancy rate (number of pregnant cows/total number of cows) of each factor. Statistical analysis: Chi-square test. Abbreviations: THI, temperature–humidity index.

**Table 5:** Binary logistic regression analysis of factors influencing pregnancy outcomes following IVP beef embryo transfer to dairy cattle.

Factor	Wald	*p*-Value	Odds Ratio	Confidence Limits (95%)
Parity	0.001	0.975	0.993	0.649–1.521
BCS	0.490	0.484	0.886	0.631–1.244
Estrus synchronization	3.038	0.081	0.860	0.725–1.019
DF volume (cm^3^)	0.406	0.524	1.096	0.826–1.454
Number of CL	0.933	0.334	0.657	0.281–1.540
CL location in the ovary	1.027	0.311	1.221	0.830–1.795
Cavitary CL	0.306	0.580	0.883	0.568–1.372
CL diameter (cm)	0.425	0.514	0.867	0.564–1.332
CL volume (cm^3^)	0.306	0.580	1.116	0.757–1.646
Cleavage rate of embryos	0.492	0.483	0.875	0.604–1.269
BL formation rate of embryos	5.212	0.022	1.450	1.054–1.996
Degeneration rate of embryos	6.169	0.013	0.737	0.580–0.938
Stage of IVP embryos	1.364	0.243	0.861	0.670–1.107
Grade of IVP embryos	10.062	0.002	0.557	0.388–0.800
Fresh/Frozen IVP embryos	1.970	0.160	1.596	0.831–3.063
THI	0.663	0.416	1.125	0.848–1.492
Transport time of embryos	0.004	0.947	0.991	0.748–1.312

Abbreviations: BCS, body condition score; DF, dominant follicle; CL, corpus luteum; BL, blastocyte; IVP, in vitro-produced; THI, temperature–humidity index.

## Data Availability

The original contributions presented in this study are included in the article. Further inquiries can be directed to the corresponding authors.

## Abbreviations

The following abbreviations are used in this manuscript:

AI	Artificial insemination
BCS	Body condition score
CI	Confidence interval
CL	Corpus luteum
COCs	Cumulus–oocyte complexes
ET	Embryo transfer
GnRH	Gonadotropin-releasing hormone
IVD embryo	In vivo-derived embryo
IVP embryo	In vitro-produced embryo
OR	Odds ratio
PGF_2α_	Prostaglandin F_2α_
PG+GN	PGF_2α_–GnRH injection
THI	Temperature–humidity index
